# Meeting the WHO physical activity guidelines and health-related quality of life of single parents in Ghana

**DOI:** 10.3389/fpubh.2025.1533284

**Published:** 2025-03-19

**Authors:** Obed Jones Owusu-Sarpong, Kabila Abass, Solomon Osei-Tutu, André Hajek, Razak M. Gyasi

**Affiliations:** ^1^Department of Geography and Rural Development, Kwame Nkrumah University of Science and Technology, Kumasi, Ghana; ^2^Department of Social Sciences, Offinso College of Education, Offinso, Ashanti, Ghana; ^3^Department of Health Economics and Health Services Research, University Medical Center Hamburg-Eppendorf, Hamburg Center for Health Economics, Hamburg, Germany; ^4^African Population and Health Research Center, Nairobi, Kenya; ^5^National Centre for Naturopathic Medicine, Faculty of Health, Southern Cross University, Lismore, NSW, Australia

**Keywords:** physical activity, quality of life, Ghana, single parents, sustainable development goals

## Abstract

**Purpose:**

Improving physical activity (PA) and health-related quality of life (HRQoL) is critically important for achieving the health-related sustainable development goal (SDG 3). However, data on the association between PA and HRQoL, particularly among single parents, are limited in low- and middle-income countries (LMICs). We examine the association between PA and HRQoL among single parents in Ghana and explore the modifying roles of sex, age, and spatial differences in this association.

**Methods:**

Data on 627 single parents were obtained through a multi-stage stratified sampling technique. PA was assessed using the International Physical Activity Questionnaire-Short Form (IPAQ-SF). The EQ-5D-3L questionnaire was used to measure HRQoL. A hierarchical Ordinary Least Squares (OLS) regression models evaluated the hypothesized associations.

**Results:**

PA was significantly associated with poor HRQoL even after accounting for all potential confounders (*B* = −0.298, SE = 0.132, *p* < 0.05). The association was further modified by sex (*B* = −0.619, SE = 0.206, *p* < 0.01) and age (*B* = −0.062, SE = 0.008, *p* < 0.001). Thus, the PA-HRQoL association was more pronounced among older and female single parents.

**Conclusion:**

Physical activity was negatively associated with poor HRQoL. Sex and age differences modified the association. Promoting PA may reduce poor HRQoL among single parents.

## 1 Introduction

Health-related quality of life (HRQoL) is conceived as a multidimensional construct encompassing an individual’s or a group’s perceived physical and mental health over time (1), including psychological, emotional, cultural orientation, and social aspects of life (2). Previous studies have investigated the determinants of HRQoL among individuals within the general population. For example, gender, place of residence, education, employment status, and smoking status have been identified as correlates of HRQoL (3–5). Studies have also examined the association between PA and HRQoL among populations (3, 6, 7). However, whether meeting the WHO PA requirements is associated with HRQoL among single parents in low- and middle-income countries is almost unknown (7).

Meeting the WHO PA guidelines is associated with numerous health benefits, including improved cardiovascular health (6), mental well-being (7), and reduced risk of chronic diseases (8) in various populations, including single parents (5). However, approximately 1 in 4 adults do not meet the global recommended levels of PA, and more than 80% of the world’s adolescent population is insufficiently physically active (5). Indeed, research suggests that meeting moderate-to-vigorous levels of PA guidelines was significantly associated with improved overall HRQoL. For instance, a cross-sectional study of 7,518 community-dwelling older Chinese revealed that PA was significantly and positively associated with each HRQoL component (9). Using a sample of 5,537 adults (40–60 years) from a representative national survey in England, Anokye et al. (10) suggest that higher levels of PA are associated with better HRQoL. However, such studies are relatively scarce in LMICs. The well-being of single parents is a vital public health concern, especially in a low- and middle-income country like Ghana, where socio-economic difficulties are widespread. The number of single parents in Ghana is rapidly increasing, primarily due to rising rates of divorce, separation, and widowhood (11). Single parents often face unique stressors that adversely affect their HRQoL (12). Understanding and addressing these challenges is crucial for creating effective public health interventions. However, there is a significant dearth of data on the association between PA and HRQoL among single parents.

Moreover, the association between PA and HRQoL may be moderated by age, sex, and spatial differences. For instance, younger single parents may be more likely to present a higher HRQoL and may be more physically active than older single parents due to the negative health effects of aging (3–13). Considering sex differences, males may have greater odds of meeting PA guidelines and the subsequent improvements in HRQoL than their female counterparts due to societal expectations and safety concerns (4, 14, 15). Again, urban environments may offer more opportunities and facilities (e.g., gyms, parks, and organized sports) for PA participation than rural areas (16–18).

Single parents are often at a higher risk of poverty and social exclusion (12). While extant studies have emphasized on PA and HRQoL in general populations, our study specifically targets single parents—a group that is often overlooked in health research in SSA, especially Ghana (5, 9). The health and well-being of single parents is critical for the socio-economic stability of families and communities (12). Therefore, the current study aims to examine the association between meeting the WHO PA guidelines and HRQoL among single parents in Ghana. We also seek to explore the potential effect modification of this association by age, sex, and rural/urban differences. The novelty of this study lies in its dual focus on a specific vulnerable group while addressing a critical aspect of public health—PA—within the context of HRQoL. The significance of this study goes beyond academic contributions. The study may potentially influence public health policies and community programs aimed at enhancing the quality of life for single parents in Ghana and similar settings. By establishing a clear link between PA and HRQoL among single parents, this research could pave the way for future studies and interventions that promote healthier lifestyles across populations facing similar socio-economic problems.

## 2 Methods

### 2.1 The survey

This study utilized multi-stage stratified sampling. The Atwima Kwanwoma District was purposefully selected based on anecdotal evidence suggesting many single parents. The district was informally divided into Atwima and Kwanwoma and further categorized into urban and rural communities based on socioeconomic characteristics and development factors such as economic activities, road conditions, and health and educational infrastructure (11). A simple random sampling method was employed to select 12 communities (i.e., four urban and eight rural communities) from 64 communities in the district (19). The names of all communities were written on slips of paper, acknowledging the informal division of the district into Atwima and Kwanwoma, which is typically split into East and West.

Using a fishbowl selection method, where names of all communities were placed in a bowl and drawn randomly (20), six communities (four rural and two urban) were chosen from Atwima, and another six communities (four rural and two urban) were selected from Kwanwoma, making a total of 12 communities (four urban and eight rural). The larger number of rural communities (8) compared to urban ones (4) was due to the higher population in urban areas. A default prevalence rate of 50% (20), equivalent to 0.5, was used to estimate the number of single parents in the district, as specific data was unavailable. Using this prevalence rate, Fisher’s sample size estimation formula was applied to determine the overall sample size for the study (21).

The minimum sample size was calculated with the following parameters: a 95% confidence interval (∝) of 1.96, a prevalence (P) of 50% or 0.5 (since the proportion of single parents aged ≥13 years is unknown), a margin of error (d) of 5% or 0.05, and considering 5% type 1 error and 15% type 2 error. Adjustments were made to account for anticipated non-responses and outliers, aiming to enhance the generalizability and robustness of the findings (22). Consequently, the final sample size was set at *N* = 627. The researchers developed a face-to-face interviewer-administered questionnaire, and five research assistants were trained to administer the questionnaire. The questionnaire is part of a broader household survey covering various topics like sleep problems, depression, anxiety, financial concerns, neighborhood attachment, self-rated health and happiness, social networks, and functional limitations. Data collection occurred over 3 months, from June to August 2023. Ethical approval was obtained from relevant authorities, and written informed consent was secured from all respondents.

Though being a purely quantitative study, a convenience sampling technique was used in reaching out to the respondents. This became necessary when we observed, during the pretesting of our research instruments, that not all households in the selected communities were single-parent households. Moreover, we met some single parents at their workplaces. Therefore, using a more appropriate sampling technique, like systematic sampling, was not feasible.

### 2.2 Ethical consideration

The study was carried out in accordance with the World Medical Association’s Declaration of Helsinki’s code of ethics for research involving human subjects. The study protocol was reviewed and approved by the Humanities and Social Sciences Research Ethics Committee (HuSSREC) at Kwame Nkrumah University of Science and Technology in Kumasi, Ghana (Ref number: HuSSREC/AP/111/VOL.1.). Additionally, written informed consent was obtained from each respondent. Respondents were assured of confidentiality and anonymity of the information they provide. They were also assured of their right to stop or refuse to respond to any question they were not comfortable with, without being penalized. Indeed, we sought permission and approval from the parents or guardians of a few minors who had less than 18 years during data collection. Moreover, in the Ghanaian context, one is considered an adult when he or she gives birth and assumes the responsibility of childcare.

### 2.3 Measures

#### 2.3.1 PA

Physical activity was evaluated using the International Physical Activity Questionnaire-Short Form (IPAQ-SF) (23), a tool that measures PA over the past 7 days as part of daily activities. Globally, the IPAQ-SF has demonstrated good validity and reliability (7, 23). The IPAQ-SF uses the following values to analyze PA: Walking = 3.3 MET (Metabolic Equivalent of Task, a unit of caloric expenditure), Moderate PA = 4.0 MET, and Vigorous PA = 8.0 MET. The continuous score for each PA category is expressed as MET-minutes per week, calculated as MET level x minutes of activity per day × days per week. Thus, the total PA MET-minutes per week is determined by summing the METs from walking, moderate activity, and vigorous activity using the formula: the sum of Walking + Moderate activity + Vigorous activity MET-minutes per week scores (23). In this analysis, the PA score was dichotomized. Those scoring less than 600 MET-minutes of moderate-to-vigorous intensity PA were classified as not meeting the recommended PA guidelines (code = 1), while those scoring 600 or more MET-minutes were classified as meeting the recommended PA guidelines (code = 2) (7). The IPAQ-SF has been validated in the Ghanaian context (7).

#### 2.3.2 HRQoL

Health-related quality of life was evaluated using the EQ-5D-3L questionnaire (20), which consists of five items covering mobility, self-care, daily activities, pain/discomfort, and restlessness. Each item was rated on a three-level scale: 1 for no problems, 2 for moderate problems, and 3 for severe problems. The total score ranges from 5 to 15, with higher scores indicating poorer HRQoL. The variable was dichotomized into high and low HRQoL, with scores from 5 to 8 representing high HRQoL and scores from 9 to 15 representing low HRQoL. This dichotomization was based on limited empirical evidence (20). Each component of the HRQoL variable was treated as a separate dependent variable and individually regressed on the independent variable. The EQ-5D-3L questionnaire has been validated in the Ghanaian context (24).

#### 2.3.3 Moderating variables

Sex, age, and spatial variations were moderating variables. Sex was considered dichotomous. A single father was given a score of 1, while a single mother scored 2. Age was measured in years (for the purpose of stratification, it was categorized into a 14–45-year group and a 46–90-year group), while residential status was also dichotomous (rural/urban).

#### 2.3.4 Covariates

Based on prior research, the analytic models were adjusted for demographic, socio-economic, lifestyle, and health-related variables (24, 25). These included socio-economic and demographic factors such as sex (male or female), residential status (rural or urban), support satisfaction, period of stay, educational attainment (measured in number of years spent in school), work status (unemployed, employed or retired), income levels (in Ghana Cedis), financial hardship (yes/no), living arrangement (i.e., living with others apart from children, yes/no), social network (Lubben Social Network Scale – 6 Item Version), economic status, food insecurity (yes/no), and religious attendance level. Additional factors included current smoking status (no = 0/yes = 1), alcohol intake (no = 0/yes = 1), depression, sleep quality, anxiety, and NHIS status (yes or no).

### 2.4 Statistical analyses

The statistical analysis was conducted using SPSS v25 (SPSS, Inc., IBM, Armonk, NY, United States). The statistical significance was determined as the *p*-value of <0.05. Sample characteristics are first displayed as stratified by the meeting of WHO PA guidelines (<600 MET min; ≥600 MET min) and HRQoL (low and high). The difference in sample characteristics was tested using Student’s *t*-tests and Chi-squared tests for continuous and categorical variables. In the second step, hierarchical OLS regression models were conducted to examine the link between meeting PA guidelines and HRQoL. We further performed interaction analysis (i.e., Sex × PA, residence × PA, and age × PA) to determine the moderation role of age, sex, and residence in the association of PA and HRQoL.

Model 1 was the unadjusted model. Model 2 included Model 1 and adjusted for age and sex. Model 3 included Model 2 and further adjusted for alcohol, residence, employment, household size, living arrangement, NHIS, education, social network, age, sex, sleep quality, depression, financial hardship, economic status, chronic conditions, and anxiety. Model 4 included Model 3 and the interaction term, PA × sex. Model 5 included Model 3 and the interaction term, PA × age. Model 6 included Model 3 and the interaction term, PA × residential status. Third, the OLS regression estimating the association between PA and the various components of HRQoL was conducted. Each component of the HRQoL variable was treated as separate dependent variables as they were individually regressed on PA and other covariates (independent variables). Lastly, OLS regression models estimating the association between meeting PA guidelines stratified by age and sex groups were also conducted. Stratification by residence was not included because the interaction analysis was not statistically significant.

## 3 Results

### 3.1 Sample characteristics

A total of 627 individuals were included in the current study. The characteristics of this analytic sample are shown in Table 1. The average age was 45.0 (SD = 14.7) years, ranging from 14 to 85 years, and 67.3% were females. The majority of the sample lived in rural communities (54.5%), were gainfully employed (71.8%), and neither smoked (97.4%) or consumed alcohol (92.5%). On average, every participant had schooled for 9.5 (SD = 4.0) years while the mean monthly income was approximately 1938.28 (SD = 904.46) Ghana Cedis (US$ 140). Moreover, the mean physical activity, social network, and number of chronic physical conditions scores were 330.79 (SD = 420.83), 8.32 (SD = 6.44), and 1.07 (SD = 1.35), respectively. A Pearson’s Chi-square test and Student *t*-test indicated that individuals who did not meet the WHO PA guidelines with <600 MET min of PA significantly had much worse scores for functional limitations (*F* = 626.60, *p* < 0.001), chronic physical conditions (*F* = 49.31, *p* < 0.001), sleep problems (*F* = 10.21, *p* < 0.01), anxiety (*F* = 8.113, *p* < 0.005), and depression (*F* = 8.02, *p* < 0.005) compared to those who met the WHO PA guidelines with ≥600 MET min of PA. Similarly, those who had low HRQoL had worse scores for functional limitations (*F* = 1033.23, *p* < 0.001), chronic conditions (*F* = 40.88, *p* < 0.001), sleep problems (*F* = 10.88, *p* < 0.001), depression (*F* = 0.12, *p* < 0.005) and anxiety (*F* = 0.11, *p* < 0.005) compared to those who had high HRQoL. Additionally, the Chi-square results showed that there was a significant association at a 5% significance level between sex and PA (*χ*^2^ = 4.076, *p* < 0.05), sex and HRQoL, (*χ*^2^ = 11.364, *p* < 0.001), residence and PA (*χ*^2^ = 4.708, *p* < 0.05). In addition, the independent *t*-test results revealed a statistically significant association between age and PA (*F* = 0.66, *p* < 0.001), age, and HRQoL (*F* = 0.031, *p* < 0.001).

**Table 1:** Demographic and health-related characteristics of the sample – overall and by meeting PA guidelines and health-related quality of life (HRQoL).

	Overall		Not meeting PA		Meeting PA		*p*-value	Statistic	Low HRQoL		High HRQoL		*p*-value	Statistic
Variable	N/M	(%)/SD	N/M	(%)/SD	N/M	(%)/SD			N/M	(%)/SD	N/M	(%)/SD		
Age (years) (M ± SD)	44.95	(14.6)	60.16	(12.36)	39.70	(11.35)	<0.001	0.66	61.35	(11.99)	41.35	(12.56)	<0.001	0.031
Sex							<0.05	4.076					<0.001	11.364
Female	422	(67.3)	98	(15.6)	324	(51.7)			92	(14.7)	330	(52.6)		
Male	205	(32.7)	63	(10.0)	142	(22.6)			22	(3.5)	183	(29.2)		
Residential type							<0.05	4.708					>0.05	0.21
Rural	342	(54.5)	76	(12.1)	266	(42.4)			60	(9.6)	282	(45)		
Urban	285	(45.5)	85	(13.6)	200	(31.9)			54	(8.6)	231	(36.8)		
Personal income (M ± SD)	1938.28	(904.46)	2118.01	(1038.8)	1876.18	(845.54)	<0.005	15.290	1914.04	(819.66)	1943.66	(922.909)	>0.05	3.233
Years of education (M ± SD)	9.47	(4.00)											<0.001	39.36
Employment status							<0.001	118.09						
Employed	450	(71.8)	76	(12.1)	374	(59.6)			62	(9.9)	388	(61.9)		
Unemployed	137	(21.9)	48	(7.7)	89	(14.2)			31	(4.9)	106	(16.9)		
Retired	40	(6.4)	37	(5.9)	3	(0.5)			21	(3.3)	19	(3.0)		
Financial hardship (M ± SD)	3.25	(2.23)	2.78	(2.36)	3.42	(2.17)	<0.01	3.78	3.62	(2.22)	3.17	(2.23)	>0.05	0.246
Smoking status							>0.05	0.413					>0.05	0.36
No	611	(97.4)	158	(25.2)	453	(72.2)			112	(17.9)	499	(79.6)		
Yes	16	(2.6)	3	(0.5)	13	(2.1)			2	(0.3)	14	(2.2)		
Alcohol status							>0.05	0.450					>0.05	0.03
No	580	(92.5)	147	(23.4)	433	(69.1)			105	(16.7)	475	(75.8)		
Yes	47	(7.5)	14	(2.2)	33	(5.3)			9	(1.4)	38	(6.1)		
Depressive symptoms (M ± SD)	15.61	(3.88)	14.70	(4.22)	15.92	(3.71)	<0.005	8.02	16.56	(3.95)	15.39	(3.84)	<0.005	0.12
Anxiety symptoms (M ± SD)	8.88	(3.82)	7.9	(3.82)	9.19	(3.82)	<0.005	8.113	9.83	(3.81)	8.67	(3.80)	<0.005	0.11
Sleep problems (M ± SD)	3.15	(0.66)	3.44	(0.59)	3.04	(0.65)	<0.01	10.21	3.59	(0.55)	3.04	(0.64)	<0.001	10.88
Chronic condition (M ± SD)	1.10	(1.35)	2.30	(1.49)	0.64	(0.98)	<0.001	49.31	2.56	(1.55)	0.74	(1.04)	<0.001	40.88
Functional limitation (M ± SD)	0.15	(0.62)	0.54	(1.13)	0.01	(0.08)	<0.001	626.60	0.02	(0.15)	0.73	(1.27)	<0.001	1033.23
Food insecurity (M ± SD)	5.36	(2.20)	4.93	(2.51)	5.52	(2.06)	<0.05	12.963	6.46	(1.78)	5.12	(2.21)	<0.001	8.16
Social network (M ± SD)	8.32	(6.44)	10.72	(6.37)	7.48	(6.26)	<0.001	0.260	7.97	(6.14)	8.40	(6.51)	>0.05	0.022
Religious attendance (M ± SD)	2.64	(0.87)	2.70	(0.79)	2.62	(0.89)	>0.05	4.80	2.72	(0.84)	2.6	(0.87)	>0.05	0.778

N is the total population; M is the mean score; SD is the standard deviation; HRQoL is the health-related quality of life; PA is physical activity. The *P*-value is based on either ordinal *χ*^2^ tests or independent *t*-test.

### 3.2 Main regression and moderation analyses

The results of the associations between meeting WHO PA guidelines and HRQoL, estimated by hierarchical OLS regression models for the pooled sample, are detailed in Table 2. The crude model (Model 1) revealed a significant negative association between PA and low HRQoL (*B* = −1.57, SE = 0.14, *p* < 0.001). After adjusting for age and sex in Model 2, the effect size decreased, indicating a weaker negative association (*B* = −0.505, SE = 0.148, *p* < 0.001). In Model 3, which adjusted for socio-demographic, socio-economic, and health-related factors, the significant negative association persisted, though the effect size was further reduced (*B* = −0.298, SE = 0.132, *p* < 0.05). Models 4 and 5 demonstrated significant moderating effects of sex (*B* = −0.619, SE = 0.206, *p* < 0.01) and age (*B* = −0.062, SE = 0.008, *p* < 0.001) on the association between PA and low HRQoL. However, the moderating role of residence in Model 6 was not significant. Further, the OLS regression analysis of PA’s impact on various HRQoL components (Table 3) showed substantial negative associations with pain/discomfort (*B* = −0.143, SE = 0.053, *p* < 0.01), usual activities (*B* = −0.311, SE = 0.040, *p* < 0.001), mobility (*B* = −0.094, SE = 0.047, *p* < 0.05), and self-care (*B* = −0.109, SE = 0.028, *p* < 0.001). Additionally, sex and age-stratified estimations of the association between PA and HRQoL (Table 4) revealed significant differences: males (*B* = −0.967, SE = 0.188, *p* < 0.001) and females (*B* = −1.330, SE = 0.147, *p* < 0.001), as well as between age groups (14–45 years: *B* = −0.395, SE = 0.193, *p* < 0.05) and (46–90 years: *B* = −1.018, SE = 0.177, *p* < 0.001). The analysis indicated that the negative association between PA and HRQoL was more pronounced among older adults and females.

**Table 2:** Hierarchical OLS regression models of the estimate of the association between meeting PA guidelines and health-related quality of life.

	Model 1		Model 2		Model 3		Model 4		Model 5		Model 6	
Variable	*B*	(SE)	*B*	(SE)	*B*	(SE)	*B*	(SE)	*B*	(SE)	*B*	(SE)
Physical activity	−1.57	(0.14)***	−0.505	(0.148)***	−0.298	(0.132)*	0.719	(0.363)*	3.010	(0.467)***	0.195	(0.321)
Age			0.058	(0.004)***	0.040	(0.005)***	0.039	(0.005)***	0.145	(0.015)***	0.039	(0.005)***
Sex (ref: male)												
Female			1.244	(0.110)***	0.757	(0.106)***	1.835	(0.374)***	0.757	(0.101)***	0.762	(0.106)***
Residential type (ref: rural)												
Urban					0.019	(0.096)	0.020	(0.096)	−0.006	(0.092)	0.599	(0.358)
Household size					−0.016	(0.022)	−0.016	(0.021)	0.030	(0.021)	−0.013	(0.021)
Years of education					−0.043	(0.013)**	−0.039	(0.012)**	−0.040	(0.012)***	−0.043	(0.012)***
Employment (ref: unemployed)												
Employed					0.078	(0.093)	0.095	(0.093)	0.078	(0.089)	0.071	(0.093)
Sleep quality					0.268	(0.080)***	0.266	(0.079)***	0.255	(0.076)***	0.272	(0.079)***
Economic status					−0.437	(0.081)***	−0.431	(0.080)***	−0.361	(0.078)***	−0.433	(0.081)***
NHIS					0.113	(0.110)	0.115	(0.109)	0.066	(0.105)	0.110	(0.110)
Social networks					−0.027	(0.008)***	−0.025	(0.008)**	−0.026	(0.008)***	−0.027	(0.008)***
Alcohol status					0.150	(0.169)	0.179	(0.167)	0.293	(0.162)	0.166	(0.168)
Financial hardship					0.075	(0.025)**	0.079	(0.025)**	0.080	(0.024)***	0.074	(0.025)**
Depressive symptoms					0.006	(0.017)	0.003	(0.017)	0.012	(0.016)	0.005	(0.017)
Chronic conditions					0.354	(0.044)***	0.369	(0.044)***	0.318	(0.043)***	0.355	(0.044)***
Anxiety symptoms					0.004	(0.018)	0.007	(0.018)	0.003	(0.017)	0.002	(0.018)
Physical activity × sex							−0.619	(0.206)**				
Physical activity × age									−0.062	(0.008)***		
Physical activity × residence											−0.332	(0.198)
Model information												
Constant	10.05	(0.25)***	3.50 (0.453)***		4.416 (0.646)***		2.57 (0.88)**		−1.925 (1.060)		3.571 (0.818)***	
Adjusted pseudo *R*^2^	0.167		0.43		0.599		0.605		0.632		0.601	
*F*-statistic	126.69		157.91		55.443		56.80		63.590		55.87	

B, Unstandardized regression coefficient; SE, robust standard error; NHIS, National health insurance scheme.

Model 1 Unadjusted model.

Model 2 included Model 1 and adjusted for age and sex.

Model 3 included Model 2 and further adjusted for alcohol, residence, employment, household size, living arrangement, NHIS, education, social network, age, sleep quality, depression, financial hardship, economic status, sex, chronic conditions and anxiety.

Model 4 included Model 3 and the interaction term, PA × sex.

Model 5 included Model 3 and the interaction term, PA × age.

Model 6 included Model 3 and the interaction term, PA × residential status.

*p < 0.05, **p < 0.01, ***p < 0.001.

**Table 3:** OLS regression models estimating the association between meeting PA guidelines and specific components of poor health-related quality of life.

Variable	Restlessness		Pain/discomfort		Usual activities		Mobility		Self-care	
	*B*	(SE)	*B*	(SE)	*B*	(SE)	*B*	(SE)	*B*	(SE)
Physical activity	0.047	(0.061)	−0.143	(0.053)**	−0.311	(0.040)***	−0.094	(0.047)*	−0.109	(0.028)***
Age	−0.004	(0.002)	0.012	(0.002)***	0.016	(0.002)***	0.025	(0.002)***	0.007	(0.001)***
Sex (ref: male)										
Female	0.208	(0.053)***	0.150	(0.046)***	0.150	(0.035)***	0.272	(0.041)***	0.040	(0.025)
Residential type (ref: Rural)										
Urban	−0.095	(0.045)*	0.066	(0.039)	−0.030	(0.030)	0.035	(0.042)	0.002	(0.021)
Sleep problems	0.128	(0.039)***	0.079	(0.034)*	0.020	(0.026)	0.016	(0.030)	0.014	(0.018)
Ethnic background	0.009	(0.019)	−0.001	(0.017)	0.000	(0.013)	0.043	(0.015)**	−0.011	(0.009)
Years of education	−0.018	(0.006)**	−0.019	(0.005)***	−0.001	(0.004)	−0.003	(0.005)	−0.001	(0.003)
Economic status	−0.075	(0.038)*	−0.113	(0.033)***	−0.081	(0.025)***	−0.096	(0.029)***	−0.085	(0.018)***
Religious attendance	0.005	(0.017)	−0.037	(0.014)*	−0.033	(0.011)**	−0.034	(0.013)**	−0.036	(0.008)***
Social networks	−0.020	(0.004)	−0.001	(0.003)	−0.006	(0.003)*	−0.004	(0.003)	−0.004	(0.002)*
Living arrangement	−0.011	(0.051)	0.072	(0.044)	−0.035	(0.034)	0.037	(0.039)	−0.039	(0.024)
Employment	0.011	(0.045)	−0.005	(0.039)	0.005	(0.030)	−0.011	(0.035)	0.034	(0.021)
Alcohol	0.146	(0.083)	0.005	(0.072)	−0.044	(0.054)	−0.026	(0.064)	−0.046	(0.038)
Depressive symptoms	0.010	(0.008)	0.000	(0.007)	0.003	(0.005)	0.01	(0.006)	0.003	(0.004)
Anxiety symptoms	0.029	(0.008)***	0.022	(0.007)**	−0.012	(0.006)*	−0.008	(0.006)	−0.012	(0.004)**
Model information										
Constant	1.064	(0.294)***	1.411 (0.255)***		1.322 (0.193)***		0.330 (0.227)		1.340 (0.137)***	
Adjusted pseudo *R*^2^	0.343		0.352		0.479		0.509		0.274	
*F*-statistic	22.62		23.46		39.066		43.801		16.607	

B, unstandardized regression coefficient; SE, robust standard error.

All models were adjusted for age, sex, alcohol, employment, residence, living arrangement, religion, social network, education, social network, age, depression, economic status, sex, and anxiety.

*p < 0.05, **p < 0.01, ***p < 0.001.

**Table 4:** OLS regression models estimating the association between meeting PA guidelines stratified by age and sex groups.

Variable	14–45 year group		46–90 year group		Males		Females	
	*B*	(SE)	*B*	(SE)	*B*	(SE)	*B*	(SE)
Physical activity	−0.395	(0.193)*	−1.018	(0.177)***	−0.967	(0.188)***	−1.330	(0.147)***
Residential type (ref: Rural)								
Urban	−0.045	(0.096)	0.288	(0.179)	0.322	(0.157)*	0.025	(0.121)
Support satisfaction	496	(0.089)***	0.137	(0.091)	0.382	(0.102)***	0.003	(0.075)
Period of stay	−0.001	(0.003)	0.015	(0.004)**	0.010	(0.004)*	0.020	(0.003)***
Years of education (ref: never)	−0.058	(0.013)***	−0.063	(0.021)**	−0.089	(0.021)***	−0.045	(0.015)**
Food insecurity	0.232	(0.030)***	0.381	(0.048)***	0.229	(0.047)***	0.334	(0.036)***
Sleep quality	0.285	(0.073)***	0.618	(0.179)***	0.321	(0.135)*	0.450	(0.097)***
Economic status	−0.075	(0.083)	−0.516	(0.167)**	−0.369	(0.166)*	−0.073	(0.103)
Social networks	0.001	(0.008)	−0.007	(0.017)	0.008	(0.012)	0.000	(0.011)
Employment	0.034	(0.110)	0.095	(0.145)	0.315	(0.171)	0.188	(0.113)
Financial hardship	−0.004	(0.025)	−0.002	(0.056)	−0.023	(0.054)	−0.018	(0.033)
Religious attendance	0.029	(0.031)	−0.138	(0.068)*	−0.113	(0.057)*	−0.022	(0.044)
Depressive symptoms	−0.031	(0.017)	0.016	(0.030)	−0.024	(0.031)	−0.041	(0.021)*
Anxiety symptoms	0.067	(0.016)***	−0.043	(0.036)	0.102	(0.035)**	−0.002	(0.022)
Model Information								
Constant	4.385	(0.618)***	6.598	(1.165)***	5.622	(0.912)***	7.145	(0.713)***
Adjusted pseudo *R*^2^	0.465		0.558		0.582		0.539	
*F*-statistic	22.563		25.531		21.106		35.756	

B, unstandardized regression coefficient; SE, robust standard error.

All models were adjusted for support satisfaction, employment, residence, period of stay, religion, education, social network, depression, financial hardship, economic status, food insecurity, sleep quality and anxiety.

*p < 0.05, **p < 0.01, ***p < 0.001.

## 4 Discussion

### 4.1 Main findings

Physical activity was significantly associated with decreased poor HRQoL, even after accounting for potential confounders. Additional analysis revealed a substantial negative association between PA and specific components of HRQoL, including pain/discomfort, usual activities, mobility, and self-care. The interaction analysis revealed a modifying effect of sex differences and age differences in the association of PA with poor HRQoL. Thus, the age and sex-stratified analysis showed that the association between PA and poor HRQoL was more pronounced among older adults and females. To our knowledge, this is the first study to provide insights into the link between PA and HRQoL among single parents in a LMIC context. This adds to our current knowledge mainly based on studies using data from high-income countries USA, China, Japan etc. Moreover, public health policies may leverage the findings of this study to create targeted health promotion programs such as community-based PA initiatives (e.g., keep fit clubs), family-oriented activities and integration of PA into health education.

### 4.2 Interpretation of findings

The results indicate a negative association between adherence to WHO PA guidelines and poor HRQoL among single parents in Ghana. Regular PA has been consistently linked to enhanced physical health, psychological well-being, and overall quality of life (3, 22, 26). Engaging in recommended PA levels appears to contribute positively to various aspects of HRQoL, including better physical functioning, reduced stress levels, and improved emotional resilience. This is consistent with previous studies investigating PA’s association with HRQoL. For instance, a study in China by Hao et al. (3) revealed PA was positively associated with HRQoL among adults. Similarly, in a large population of Tehranian adults, Jalali-Farahani et al. (26) found levels of PA were significantly associated with most subscales of HRQoL in both men (*p* < 0.05) and women (*p* < 0.01).

The association between PA and HRQoL may be explained by a range of physiological, psychological, and socio-economic mechanisms (3). Physiologically, regular PA strengthens the heart, enhances blood circulation, and lowers the risk of heart disease (10). PA may improve cardiovascular health by enhancing heart and lung function, reducing blood pressure, improving blood lipid profiles and thereby enhancing health-related quality of life (6). Regular PA may also help regulate blood sugar levels and improve insulin sensitivity, reducing the risk of type 2 diabetes and metabolic syndrome (8). Furthermore, PA builds muscle strength and endurance, making everyday activities easier, leading to high HRQoL. Additionally, regular PA aids in maintaining a healthy weight, which is associated with a reduced risk of chronic physical conditions such as diabetes and hypertension (9).

The link between regular PA and psychological health are equally compelling. PA may reduce symptoms of anxiety and depression through the release of endorphins, commonly known as “feel-good” hormones (7, 10). PA serves as a natural way to relieve stress, aiding individuals in managing daily challenges more effectively (7). Regular physical activity may mitigate the adverse effects of stress and improve mental health outcomes, which are particularly relevant for single parents who often face elevated levels of stress due to economic pressures, child-rearing responsibilities, and social isolation (12, 26, 27). Cognitively, PA may boost memory and learning abilities, which are essential for sustaining a good quality of life (10). This brain boost is partly due to its influence on mood-regulating neurotransmitters like serotonin and dopamine (25). Furthermore, participating in group fitness classes or team sports fosters social interactions, build friendships and support networks which provide a sense of belonging (26). That sense of belonging and connection is invaluable to HRQoL (26, 27).

The moderation analysis revealed that age and sex variations emerge as critical moderators that shape the magnitude and manifestation of the association between PA and HRQoL. The negative interaction term of age variations on the association of PA with poor HRQoL shows that the association was much stronger among younger single parents (14–45 years) compared with those aged (46 and above). Thus, as one ages, the effect of PA on HRQoL decreases. Age plays a crucial moderating role in the relationship between PA adherence and HRQoL. Younger single parents, typically more active and energetic, may experience greater benefits in terms of improved mood, energy levels, and stress reduction from regular physical activity, thereby leading to higher HRQoL (3). Moreover, age-related declines in muscle mass (sarcopenia), bone density (osteoporosis), cardiovascular health and the fear of injury can make certain activities more difficult or even dangerous (28). Beyond the physical, older adults might grapple with the loss of peers, chronic health conditions impacting their mental well-being, or even societal ageism (stereotypes), all of which can affect their willingness to embrace an active lifestyle (3). This aligns with existing studies. For example, Bélanger et al. (28) suggest that PA adherence is greater for young adults (16–24 years: men = 27%; women = 21%) than older adults (65+ years: 12%; 11%). Etxeberria et al. (13) also found that the oldest old had a poorer HRQoL in both physical and mental dimensions. However, an interaction analysis from Hao et al. (3) indicated that the relationship between PA and HRQoL was significantly different across young, middle-aged, and older Chinese adults (*p* < 0.05), revealing that older adults with the sufficient PA (coefficient = 0.090, 95%CI: [0.081, 0.100]) and active PA (coefficient = 0.057, 95%CI: [0.043, 0.072]) had significantly higher HRQoL compared with young and middle-aged groups.

The interaction analysis also revealed a negative modifying effect of sex differences in the association of PA and low HRQoL. The results suggest that PA has a differential association with HRQoL based on sex. The current study reveals that while physical activity positively influences HRQoL in both sexes, the magnitude of this benefit is higher in males. Empirical studies show that males and females have different physiological responses to physical activity, which might influence how physical activity affects their HRQoL (15). Biologically, women undergo hormonal changes throughout their lives. For women, hormonal fluctuations throughout their lifespan – menstruation, pregnancy, and menopause – can significantly influence energy levels, mood, and physical performance, thus impacting their perception of PA benefits (4). Furthermore, differences in body composition – women generally having a higher percentage of body fat and lower muscle mass than men – can influence which types of activity they find most beneficial or enjoyable (14). Again, cultural and societal expectations regarding physical activity may differ between sexes as males might be more encouraged or expected to engage in physical activity and thus might derive greater psychological and social benefits from such engagement (7). Furthermore, the type and intensity of physical activity that males and females typically engage in may differ, potentially leading to different impacts on HRQoL. For instance, males might participate more in high-intensity or team sports, which could have stronger effects on improving HRQoL (14). On the other hand, Ji et al. (15) suggest that women, compared with men, derived greater gains in all-cause and cardiovascular mortality risk reduction from equivalent doses of leisure-time PA.

### 4.3 Implications for policy, practice, and research

The findings from this study, pending future longitudinal research, highlight the need for policies and interventions that promote the development of age and sex-specific PA programs. For example, younger single parents may benefit from high-intensity and time-efficient exercises, while older single parents might need low-impact and flexibility-focused activities like stretching, dancing and swimming. Programs may also consider the different time constraints, safety concerns, and social support systems needed for both males and females. Economic barriers may hinder single parents from engaging in regular PA. Policies by state and non-governmental agencies that provide subsidies or financial incentives for gym memberships, fitness classes, or sports participation may enhance access and adherence to PA. Again, health practitioners may promote PA options that may be easily integrated into the daily routines of single parents. Recommendations could include walking, home-based exercises, or family-oriented PA. Longitudinal research may provide insights into the long-term effects of meeting WHO PA guidelines on the HRQoL of single parents, highlighting the sustainability and effectiveness of various interventions.

### 4.4 Strengths and limitations

This study is among the few to investigate the relationship between PA and HRQoL, and it is the first to explore how age and sex differences moderate this association, specifically among single parents in Ghana. Given the limited information on PA among single parents in Ghana, the findings from this study could serve as a valuable baseline for future research. The key variables in the study were assessed using validated scales known for their robust reliability and content validity. Additionally, various potential confounders were controlled to enhance the validity of the results. Despite these strengths, the study has limitations. The cross-sectional design of the research limits the ability to establish causal relationships. Therefore, future studies may use a longitudinal design to draw causal conclusions. The reliance on retrospective self-reporting in the questionnaire may introduce recall and reporting biases. Again, future studies may consider clinical assessments of HRQoL to strengthen the validity of the findings. Nonetheless, self-reporting remains a practical method for capturing respondents’ subjective evaluations of their HRQoL.

## 5 Conclusion

PA was negatively associated with poor HRQoL among single parents in the LMIC contexts and the association was significantly modified by age and sex differences. These findings highlight the importance of considering age and sex differences when designing and implementing PA interventions to improve HRQoL. Tailoring interventions to address the specific needs and responses of various age and sex groups may enhance their effectiveness.

## Data Availability

The raw data supporting the conclusions of this article will be made available by the authors, without undue reservation.
